# Upgrading Electricity Generation and Electromagnetic Interference Shielding Efficiency via Phase‐Change Feedback and Simple Origami Strategy

**DOI:** 10.1002/advs.202206835

**Published:** 2023-03-22

**Authors:** Xinpeng Hu, Bingqing Quan, Chuanbiao Zhu, Haoye Wen, Mengjie Sheng, Shuang Liu, Xiaolong Li, Hao Wu, Xiang Lu, Jinping Qu

**Affiliations:** ^1^ Key Laboratory of Material Chemistry for Energy Conversion and Storage Huazhong University of Science & Technology Ministry of Education Wuhan 430074 P. R. China; ^2^ Hubei Engineering Research Center for Biomaterials and Medical Protective Materials Huazhong University of Science & Technology Wuhan 430074 P. R. China

**Keywords:** electromagnetic interference shielding, light‐thermal‐electric conversion, phase‐change feedback, simple origami strategy, upgrading electricity generation

## Abstract

Developing ultimate electromagnetic interference (EMI) shielding materials that can simultaneously upgrade the quality of generated electricity and the light‐thermal‐electric conversion efficiency based on traditional thermoelectric devices is crucially desired. Herein, a series of flexible multilayered phase change films (PCFs) is developed by a simple and novel origami strategy. The PCFs are first reported to improve the light‐thermal‐electric conversion efficiency by as high as 11.3%. Simultaneously, the PCFs could significantly upgrade the generated electricity on average voltage (27.3%), average current (23.8%), and lasting power outputs by 2010 times from microwatts to milliwatts. Besides, the EMI shielding efficiency of PCFs could be tuned from 39.2 to 71.9 dB by the origami process, the wide‐range EMI shielding performance could be suitable for varying occasions. Overall, this work provides a promising solution for both the preparation of multifunctional materials, high‐efficiency solar energy harvesting and upgrading electricity generation, which shows broad application prospects in EMI shielding, energy storage, and conversion.

## Graphical abstract

The origami process is employed and proved to be efficient to tune the EMI shielding efficiency and improve the light‐thermal‐electric conversion efficiency with the phase‐change‐feedback strategy.

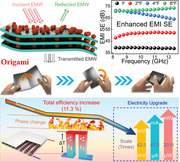

## 1 Introduction

With the global restrictions on carbon emissions, solar energy has attracted widespread attention due to its huge amount and easy access.^[^ ^1^ ^]^ The utilization of solar energy, especially the direct conversion from solar energy into electricity, has aroused increasing interest in research and industrial applications. There are two main approaches to converting solar energy into electricity. The first is to directly convert solar energy into electricity through photovoltaic cells such as perovskite solar cells (PSCs),^[^ ^2^ ^]^ organic solar cells,^[^ ^3^ ^]^ etc. Another method is to convert solar energy into heat, and then convert the heat into electricity through thermoelectric devices.^[^ ^4^ ^]^ This method is simple and convenient, which only requires a temperature gradient to generate electricity. Therefore, it has been widely used in aerospace, batteries, and the human body, etc.^[^ ^5^ ^]^

Based on light‐thermal‐electric conversion, the generation of high‐grade electricity demands both high conversion efficiency and a sufficient temperature gradient. Over the past 30 years, the thermoelectric community has witnessed unprecedented achievements in improving traditional thermoelectric materials.^[^ ^6^ ^]^ Whereas, the energy harvesting still suffers from low total thermoelectric conversion efficiency, the thermoelectric conversion efficiencies of commercial devices are always no more than 8%.^[^ ^7^ ^]^ The main attention is focused on thermoelectric materials rather than the whole thermoelectric system. Given that the improvement of thermoelectric conversion efficiency is difficult on materials, the discovery of a new strategy to improve the total conversion efficiency from energy source to electricity could be a promising direction.

On the other hand, a sufficient temperature gradient always requires fast heat charge of hot sources, which demands efficient and rapid harvest of solar energy.^[^ ^8^ ^]^ Since the natural solar light is always intermittent, the grade and stability of generated electricity are always not ideal.^[^ ^9^ ^]^ Therefore, materials with high light‐to‐thermal efficiency and lasting energy supply to upgrade generated electricity based on traditional thermoelectric devices are highly demanding.

In addition, thermoelectric devices are always employed to power advanced low‐energy electronics (such as energy‐autonomous IoT devices, flexible electronics).^[^ ^10^ ^]^ However, due to the rapid development of communication technologies, these devices are always exposed to huge electromagnetic interference (EMI) and prone to experience performance, and reliability degradation. Therefore, the hot source materials simultaneously possessing high EMI shielding efficiency have increasingly become a research hotspot.^[^ ^11^ ^]^ Whereas, the preparation of such multifunctional material often involves complex fabrication processes. There are many efforts devoted to fine structures like segregated structures and aerogel. For example, Yan et al.^[^ ^12^ ^]^ fabricated a series of reduced graphene oxide (rGO)/polystyrene (PS) composites via high‐pressure solid‐phase compression molding. The composites with segregated structures possess an EMI shielding effectiveness of 45.1 dB with only 3.47 vol% rGO loading. Zhao et al.^[^ ^13^ ^]^ constructed a highly conductive MXene/rGO aerogel through hydrothermal assembly, directional freezing and freeze‐drying process. The resulting aerogel exhibited EMI shielding effectiveness of more than 50 dB at a low MXene content of 0.74 vol%. These methods require ultrahigh pressure, complicated process or chemical reactions, which are not facile in practical production. Another method is to prepare multifunctional composites by layer‐by‐layer assembly. Gu et al.^[^ ^14^ ^]^ fabricated a series of silver nanowire decorated leather (AgNW/leather) nanocomposites with layered structures via the vacuum‐assisted filtration technique. While the AgNW network endowed the composites with excellent EMI shielding efficiency, the composites simultaneously acquired considerate thermal management properties in cold weather. Our previous work reported the fabrication of multilayered polypropylene (PP)/carbon nanotube (CNT)/paraffin wax (PW)/ethylene propylene diene monomer (EPDM) films.^[^ ^15^ ^]^ The nanocomposite exhibited high EMI shielding efficiency of 57.2 dB and ideal icing removal performance. However, when the composites contain multiple layers, this fabrication process takes a lot of time.^[^ ^16^ ^]^ The simple fabrication of multifunctional nanocomposites could be beneficial to both environment and production efficiency.^[^ ^17^ ^]^

**Figure 1:**
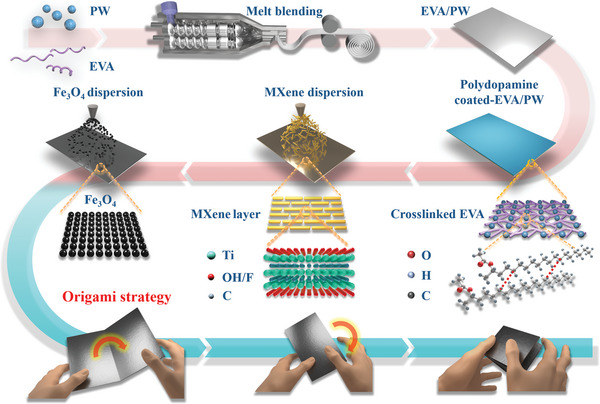
Schematic diagram for the preparation of multilayered PCFs by origami process.

Based on the above problems, for the first time, we employed a simple origami strategy to prepare flexible multilayered phase change films (PCFs). With the flexible ethylene vinyl acetate/paraffin wax (EVA/PW) film as the phase change layer, and MXene/Fe_3_O_4_ bilayer as the photothermal and electromagnetic shielding layer, a series of PCFs has been produced (**Figure** **1**). The PCFs maintain considerate flexibility after multiple folding, and can supply stable and rapid heat charge in the light‐thermal‐electric conversion. As a result, the power output reaches as high as 138 mW, whose density (55.2 W m^−2^) is notably high in the literature. For the first time, PCFs were found successfully to upgrade the generated electricity due to the phase change process. And the total efficiency of light‐thermal‐electric conversion efficiency has been increased by 11.3% based on the traditional thermoelectric devices. The discovery is vital for the effective utilization of solar energy and thermoelectric conversion. On the other hand, the EMI shielding efficiency of the PCFs can be adjusted from 39.2 to 71.9 dB by a simple origami process. Overall, this article provides a new strategy for the preparation of multifunctional materials, and the employment of PCFs as light harvesters has significantly upgraded the generated electricity and light‐thermal‐electric conversion efficiency. The PCFs show broad application prospects in EMI shielding, energy storage and conversion.

## 2 Results

### 2.1 Morphology and Structure Characterizations of Single‐Layered PCFs

**Figure 2:**
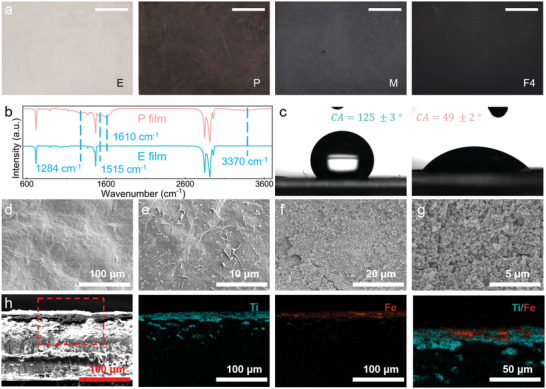
Property and structure characterizations of flexible single‐layered and multilayered PCFs by origami process. a) The pictures of appearances of E, P, M, and F4 (with 4 wt% Fe_3_O_4_) films (scale bar: 1 cm). b) FTIR spectra of E films before and after polydopamine coating. c) Contact angles of E films before and after polydopamine coating. d) SEM picture of MXene coating. e) Magnified SEM picture of MXene coating. f) SEM picture of Fe_3_O_4_ coating. g) Magnified SEM picture of Fe_3_O_4_ coating. h) EDS spectra of F4. The data in figure c) are obtained from at least three measurements for each set of samples and presented in form of mean ± standard deviation.

The preparation of Fe_3_O_4_@MXene@polydopamine‐coated EVA/PW (F) PCFs involves the dopamine polymerization coating and two steps of filler spray coatings (Figure 1). After being dived into 48 h dopamine polymerization, the color of polydopamine‐coated EVA/PW (P) film turned from white (EVA/PW (E)) to deep brown (**Figure** **2**a). The change of color results from the successful coating of polydopamine, which is known for dark color.^[^ ^18^ ^]^ The FTIR was conducted to characterize the surface structural changes of P film. After the dopamine polymerization, there were some new peaks existing at 1284, 1515, 1610, and 3370 cm^−1^ (Figure 2b). These new signals are derived from the stretching vibration of phenolic C—O, shearing vibration of N—H, stretching vibration of aromatic ring and bending vibration of N—H, stretching vibration of phenolic O—H and N—H, respectively, all of which belongs to the polydopamine.^[^ ^19^ ^]^ These results demonstrate the successful coating of polydopamine on the E film. As a result, the polydopamine coating has significantly improved the hydrophilicity of the P film (Figure 2c). Before the polydopamine coating, E film displayed a contact angle of 124.8° due to the non‐hydrophilic nature of both EVA and PW. After the dopamine polymerization, the contact angle decreased to 49.1°, which is beneficial to the adhesion of hydrophilic substances.

Under the assistance of polydopamine coating, the MXene and Fe_3_O_4_ were revealed to be successfully coated onto the P film. The MXene appeared rather homogeneous on the surface (Figure 2d). In a magnified picture, the sprayed MXene sheets were assembled into a continuous layered structure without obvious aggregation and evenly cover on the surface of P film (Figure 2e). Similarly, the Fe_3_O_4_ dispersion was sprayed coating onto the MXene@polydopamine‐coated EVA/PW (M) film and the Fe_3_O_4_ spherical nanoparticles have totally covered the MXene layer (Figure 2f,g). In addition, the Fe_3_O_4_ spherical nanoparticles were arranged in a uniform and even style, which agrees with the structure design of F film. The EDS analysis was employed to verify the distribution of MXene and Fe_3_O_4_. Both the Ti and Fe elements are distributed in a continuous layered style. In a magnified image, the Fe element is dispersed beyond the Ti element. It is fully consistent with the initial design. Thereby, the well‐defined layered‐structured F PCFs were proved to be fabricated.

### 2.2 Property and Structure Characterizations of Flexible Single‐Layered and Multilayered PCFs by Origami Process

**Figure 3:**
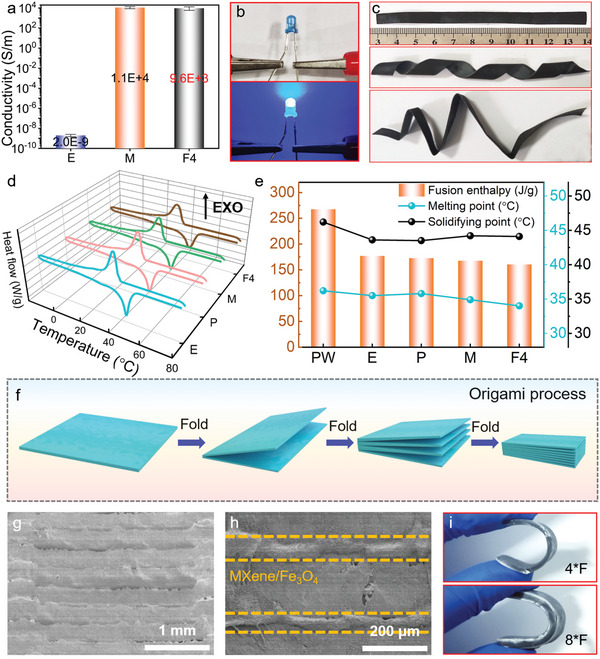
Property and structure characterizations of flexible single‐layered and multilayered PCFs by origami process. a) The electrical conductivity of E, M, and F4 films. b) The conduction tests of P and F4 films. c) Flexibility tests of F4 films. d) DSC curves of PCFs. e) Phase change parameters of PW and PCFs. f) Origami process for multilayered PCFs. g) The SEM morphology of 8*F4. h) The magnified SEM morphology of 8*F4. i) Bending tests of 4*F4 and 8*F4. The data in figure a) are obtained from at least three measurements for each set of samples and presented in form of mean ± standard deviation.

With the continuous layered MXene coating introduced, the electrical conductivities of M and F films were significantly improved (**Figure** **3**a). M and F films acquired electrical conductivities as high as 1.1 × 10^4^ and 9.6 × 10^3^ S m^−1^, respectively. Using as a conductor, the F film (bottom) worked well in a circuit to drive the LED while the P film could not (Figure 3b, Figure S1, Supporting Information). In addition, the F film exhibited excellent flexibility, it could be folded into spiral and Zigzag shapes without brittle failure (Figure 3c). The flexibility comes from the crosslinked skeleton of EVA^[^ ^20^ ^]^ (Figure S2a, Supporting Information). The PW is well constrained by the EVA skeleton, which forms a continuous network (Figure S2b, Supporting Information). The network could well support the PCFs, thereby endowing the PCFs with excellent flexibility.^[^ ^21^ ^]^

The phase change properties of PCFs were characterized by differential scanning calorimetry (DSC) (Figure 3d, Figure S3, Supporting Information). The melting and solidifying point of PW are 46.2 and 36.2 °C, respectively. And the curve of E has changed significantly as the EVA was introduced, the melting and solidifying point decreased to 43.6 and 35.5 °C, respectively (Figure 3e). This is because the crosslinked EVA skeleton constrains the crystallization of PW.^[^ ^22^ ^]^ However, the introduction of polydopamine, MXene, and Fe_3_O_4_ coating hardly influenced the phase change properties of PW as the curves are almost the same. The three PCFs have nearly the same solidifying and melting points (35.8 and 43.5 °C for P, 34.9 and 44.2 °C for M, 34.0 and 44.1 °C for F4). Besides, the fusion enthalpy for PW is 267.5 J g^−1^. With the introduction of EVA, polydopamine, MXene and Fe_3_O_4_ coating, the values decreased to 177.1, 172.8, 167.7, and 160.5 J g^−1^, respectively, which decrease with the reduced PW contents. These results indicate that PW plays the dominant role in energy storage of PCFs.^[^ ^23^ ^]^ The EVA and functional layers only serve as the supporting skeleton, and the introduction of coating does not affect the phase change characteristics of PW. However, all of the enthalpies of PCFs are above 160 J g^−1^, ensuring a large amount of energy can be stored during the charging process, which is competitive on varying occasions. Besides, the DSC curves were observed to be with insignificant attenuation after 10 thermal cycles (Figure S4, Supporting Information). The PCF shows high enthalpy, excellent phase change stability and reusability for long‐term service.

With such notable flexibility and excellent phase change parameters, the F4 films were folded by varying times to fabricate multilayered PCFs (Figure 3f). The obtained 8*F4 PCF possessed a typical multilayered structure as shown by the fracture morphology (Figure 3g). There were no obvious defects in the structure and each layer obtained almost same thickness. In addition, the simple origami process hardly changed the structure of PCF, the MXene and Fe_3_O_4_ functional layers still assembled in a continuous layer (Figure 3h). Simultaneously, the multilayered PCFs presented considerate flexibility in which it could be bent by a large angle (Figure 3i). It is inherent from the excellent flexibility of F film. And the multilayered PCFs could fully keep the initial shapes at both room temperature and beyond the melting point (Figure S5, Supporting Information). At room temperature, both the PW and PCFs could keep the initial shapes. After being put into the oven at 60 °C, the PW melted and deformed. The PCF experienced the phase change process obviously while it could still keep its shape steady. The PCFs exhibited excellent reusability and shape stability.

### 2.3 Enhanced and Tunable EMI Shielding Efficiency of Multilayered PCFs by Origami Process

**Figure 4:**
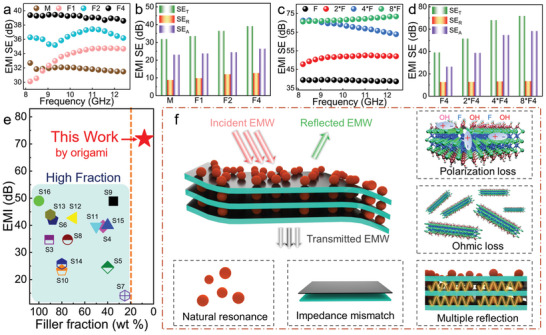
EMI shielding efficiency and mechanism of PCFs. a) EMI shielding efficiency of single‐layer F films. b) EMI shielding efficiency of F films with varying layers by origami strategy. c) EMI shielding efficiency summarization of single‐layer F films. d) EMI shielding efficiency summarizations of F films with varying layers by origami strategy. e) Comparison with other EMI shielding materials with different contents of fillers in previous studies. f) Illustration EMI shielding mechanism of PCFs.

With highly conductive and continuous MXene layer, the flexible PCFs show great prospects for EMI shielding applications. **Figure** **4**a shows the EMI shielding efficiency of single‐layered PCFs in X‐band (8.2–12.4 GHz) with varying Fe_3_O_4_ contents. It can be seen that the EMI shielding efficiency of the M film is 31.9 dB. With the increase of Fe_3_O_4_ content to 4.0 wt%, the EMI shielding efficiency gradually increases to 39.2 dB. The calculated SE_A_ and SE_R_ verify the impact of Fe_3_O_4_ on EMI shielding efficiency (Figure 4b). The introduction of Fe_3_O_4_ promotes not only the improvement of SE_A_ but also SE_R_, which indicates that the addition of Fe_3_O_4_ enhances both the absorption and reflection of electromagnetic waves.^[^ ^24^ ^]^ This is because Fe_3_O_4_ possesses higher electrical conductivity than the P film, thus improving the reflection of electromagnetic waves.^[^ ^25^ ^]^ In addition, Fe_3_O_4_ magnetic nanoparticles can reduce electromagnetic waves through the natural resonance in the GHz band.^[^ ^26^ ^]^

The EMI shielding efficiencies of multilayered PCFs by simple origami process are shown by Figure 4c. As mentioned before, the single‐layered F4 possesses an EMI shielding efficiency of 39.2 dB. After the origami process, 2*F4, 4*F4, and 8*F4 obtain EMI shielding efficiencies of 51.6, 68.1, and 71.9 dB, respectively, which increase with the origami process. Remarkably, the EMI shielding efficiencies of PCFs could be tuned in the wide range from 39.2 to 71.9 dB, showing great suitability on varying occasions. In addition, the PCF acquires superior EMI shielding efficiency (71.9 dB) with a low fraction (8 wt%) of filler. Comparing with the previously reported MXene‐based EMI shielding nanocomposites with varying contents of MXene (Figure 4e, Table S1, Supporting Information), our PCFs overweigh most EMI shielding nanocomposites with a low fraction of filler, for high and wide‐range tunable EMI shielding efficiency. On the other hand, the SE_A_ and SE_R_ were calculated to characterize the effect of origami process (Figure 4d). While SE_R_ hardly shows any changes, the SE_A_ shows obvious improvement from 26.4 to 58.4 dB, which are deduced by the increasing layers of PCFs. Figure 4f illustrates the process of electromagnetic waves transmitted through the multilayered PCFs. When the initial incident electromagnetic waves contact with Fe_3_O_4_ magnetic nanoparticles on the PCF upper surface, a part of electromagnetic waves is lost due to the natural resonance. As electromagnetic waves contact with MXene layer, the majority of the electromagnetic waves are reflected back into the Fe_3_O_4_ because of the large impedance mismatch between Fe_3_O_4_ and the MXene.^[^ ^27^ ^]^ During this period, natural resonance occurs and electromagnetic waves are lost again. The remaining electromagnetic waves would experience polarization loss as it interacts with the functional groups (OH, F, etc.) on the MXene surface.^[^ ^28^ ^]^ Then, they will interact with high‐density electron carriers when the electromagnetic waves pass through the MXene layer, leading to drastic attenuation of electromagnetic waves.^[^ ^29^ ^]^ Besides, the electromagnetic waves experience massive ohmic losses between a large number of parallel MXene sheets in the MXene layer.^[^ ^30^ ^]^ As the surviving electromagnetic waves enter the layered structure of the PCFs, they would go through plenty of multi‐reflections.^[^ ^31^ ^]^ In the whole transmittance process, the repeated reflection and scattering greatly prolong the pathway of electromagnetic waves. And the lost energy of electromagnetic waves converts into heat in the form of microcurrent, therefore significantly enhancing the EMI shielding efficiency.

In addition, Ti element in MXene nanosheets is prone to be oxidized to form TiO_2_,^[^ ^32^ ^]^ which would reduce the electric conductivity of MXene. As shown before (Figure 3h), the MXene layer located between the polydopamine‐coated EVA/PW and Fe_3_O_4_, and the Fe_3_O_4_ totally covered the MXene layer in PCFs. The layered structure could protect MXene from oxidation because of the contact with oxygen in the air. This would extend the service time of the PCFs.

### 2.4 Enhanced and Rapid Light‐To‐Thermal Conversion of Multilayered PCFs by Origami Process

**Figure 5:**
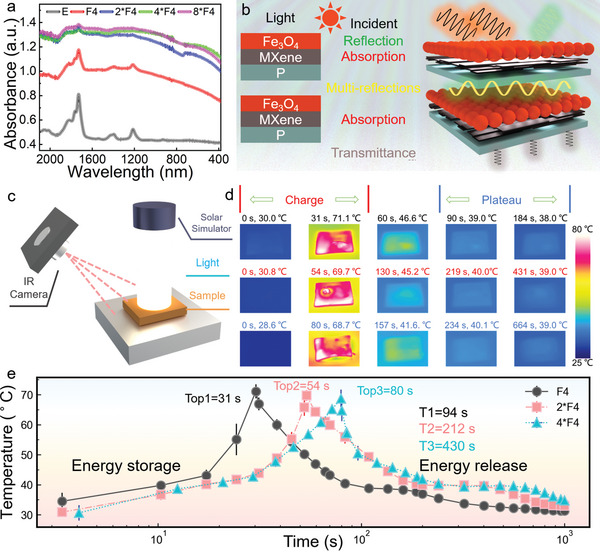
Enhanced and rapid light‐to‐thermal conversion of multilayered PCFs by origami process. a) Light absorption curves of PCFs. b) Illustration of light transmittance process through PCFs. c) Set‐up for the PCFs to harvest light. d) In‐time pictures of PCFs (F4, 2*F4, and 4*F4) under simulated solar light (one sun). e) Temperature curves of PCFs (F4, 2*F4, and 4*F4) under simulated solar light (one sun).

Being applied to light‐thermal‐electric conversion, efficient and rapid light harvest is essential. The P film is not highly capable of light harvest as it exhibits a low light absorption efficiency in the whole UV–vis‐IR wavelength range, its average light absorbance locates at 0.55 a.u (**Figure** **5**a, Figure S6, Supporting Information). Comparably, after introducing the MXene and Fe_3_O_4_, the light absorption has been significantly improved in the whole range of wavenumber attributing to high light absorption performance of MXene.^[^ ^33^ ^]^ The average absorbance of F4, 2*F4, 4*F4, and 8*F4 are 0.98, 1.23, 1.27 and 1.28 a.u., respectively, which are much higher than that of P film. Overall, the light absorbance increases by the origami process. And the light absorption increases rapidly in the first two folding processes and keeps almost stable at 4*F4 to 8*F4. This is because that 4*F4 is already able to absorb almost all the light. The transmittance parameters could assistant explain the results (Figure S7, Supporting Information). For the 4*F4, the transmittance comes to close to 0, and there is almost no light transmitting through the film. With the layers increased to 8*F4, not much light could be absorbed. Therefore, there is no further significant improvement in the absorbance from 4*F4 to 8*F4. The PCF has obtained superior light absorption performance. Figure 5b represents the light absorption process of PCFs. The light transmitting through the F4 needs to experience reflection and absorption of functional coatings including polydopamine, MXene and Fe_3_O_4_. With the layers increasing, the light will go through multi‐reflections between the layers and more absorption by the functional coatings. In this regard, with the layers increasing, there is more light being absorbed and less transmitted. However, as the PCFs come to four layers, there is nearly no light transmitted from the 4*F4 and the light absorption is almost saturated, revealing that the 4*F4 has acquired excellent light absorbance. The results demonstrate the origami strategy is an efficient method to enhance the light harvest performance.

Obtaining excellent light absorption performance, the light‐to‐thermal conversion test was launched. The pictures as well as time‐temperature curves of varying PCFs under simulated solar light were recorded (Figure 5c). The curves could aid to compare the light harvest performances of PCFs. In addition, the corresponding videos have been provided in the Supporting Information (Videos S1–S3, Supporting Information). As the PCFs were heated to 70 °C and the light was cut off, the F4 film spent 31 s to increase its temperature from 30 to 70 °C, exhibiting excellent light harvest ability (Figure 5e). The 2*F4 and 4*F4 PCFs spent 54 and 81 s to about 70 °C, respectively. The light‐to‐thermal process could be described by the following Equations (1) and (2):

(1) $$Q\;=\;m\left(\Delta H\;+c_{p}\Delta T\right)$$

(2) Q=PΔt

Where Q represents the absorbed heat in the process, P is the light‐to‐thermal output power. Δ*H*, *c*_p_, *m* are the enthalpy, specific heat capacity and the weight of PCFs, respectively. Δ*T* is the temperature change and Δ*t* is the time spent in the heating process. Therefore,

(3) $$P\;=\;\left(\Delta H\;+c_{p}\Delta T\right)\frac{m}{\Delta t}$$

As the Δ*H*, *c*_p_, and Δ*T* are constant values, the value of $\frac{m}{\Delta t}$ could reflect the power input of PCFs (Equation (3)). The time of 2*F4 to 70 °C is less than two times of F4 and 4*F4 is less than two times that of 2*F4, suggesting that the power inputs by PCFs increase with the origami process (4*F4 > 2*F4 > *F*4). Therefore, the reduced times originate from the improved light‐to‐thermal conversion efficiency of PCFs. In addition, both among the light on and off stages, the PCFs show obvious plateaus owing to the phase change process. The energy release stage shows that the plateau of 4*F4 is two times and four times longer than 2*F4 and F4, simultaneously proving the enhanced light harvest ability of 4*F4 and its significant energy storage performance (Figure 5d). The origami strategy was proved to be an efficient method to boost both EMI shielding and solar energy harvest. The method could be a universal method to fabricate high‐performance multifunctional composites.

### 2.5 Phase‐Change‐Feedback Improved Light‐Thermal‐Electric Conversion Efficiency and Electricity Upgrade

**Figure 6:**
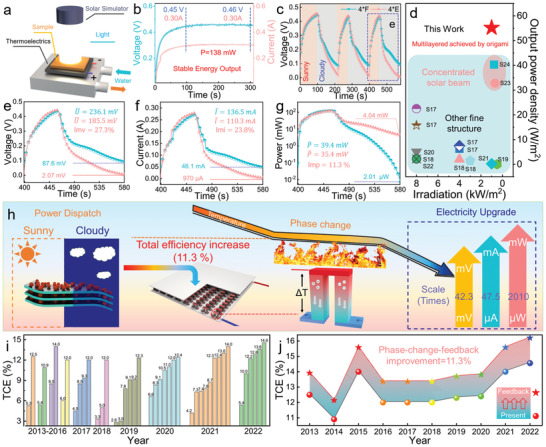
Phase‐change‐feedback improved light‐thermal‐electric conversion efficiency and electricity upgrade. a) Designed device for light−thermal−electric conversion. b) Output voltage and current of LTED based on 4*F with the stable light (one sun). c) Output voltage of LTED based on 4*F and 4*E with intermittent light (sunny light input: one sun). d) Comparison with other PCM‐supported LTEDs in previous studies. e) Output voltage of LTED based on 4*F and 4*E in a cycle. f) Output current of LTED based on 4*F and 4*E in a cycle. g) Output power of LTED based on 4*F and 4*E in a cycle. h) Illustration of improvement effect of phase‐change feedback on electricity generation. i) Summarization of thermoelectric conversion efficiency (TCE) of typical thermoelectric materials from 2013 to 2022. j) The calculated thermoelectric conversion efficiency based on phase‐change‐feedback strategy and previously reported high‐performance thermoelectric materials.

Being identified as an excellent solar energy harvester with high phase change enthalpy, the 4*F4 was employed as the heat source in the light‐thermal‐electric conversion (**Figure** **6**a). The PCF and light thermoelectric device (LTED) were directly put under the simulated solar light. As shown by Figure 6b, once the light was on, there was electricity generated by the LTED and the electricity topped at about 100 s. The generated electricity is originated from the temperature gradient between PCFs and the cooling pan. At this stage, the voltage and current keep stable at 0.46 V and 0.30 A, respectively. The stable electricity output comes from the stable heat input provided by the PCF.^[^ ^34^ ^]^ With the stable light input, the power output kept extremely stable at ≈138 mW. Worth mentioning, the calculated power density is significantly high, which could even achieve as high as 55.2 W m^−2^. As for previously reported phase change composites, our PCFs merit the fast but uniform heat‐transfer characteristic and rapid harvest of solar energy, high but durable electricity output, which is distinguished from most similar‐type LTEDs reported (Figure 6d, Table S2, Supporting Information).

As solar light always fluctuates with the weather, the electricity generated by the LTED is always not stable enough and its grade is low, the upgrade of this kind of electricity in the initial stage is essential. In this regard, simulated light with an intermittent light mode was applied to the PCF‐based LTED (Figure 6c). Similarly, the electricity rapidly increased with the light exposure initially. As the illumination was cut off, the generated electricity quickly decreased. Clear platforms for the changes of current and voltage were observed, the platforms result from that energy stored by the PCF is released to extend the power generation. On the contrary, once the solar light simulation was cut off, both the voltage and current of the control sample (4*[Fe_3_O_4_/MXene/polydopamine‐coated EVA film] (4*E4)) decreased with no decay, the grade of generated electricity was downward. The U‐I curves (Figure 6e,f) simultaneously reveal that the PCFs are able to significantly improve the grade of generated electricity on current, voltage and power output. Detailly, the average voltage and current generated by PCF‐based LTED are 236.1 mV and 136.5 mA, respectively, while those of 4*E4‐based are only 185.5 mV and 110.3 mA, respectively. Both the average voltage and current outputs achieve significant improvements ( $Im_{x}=\frac{\Delta x}{x}\;,\;$Imv: 27.3% and Imi: 23.8%). Besides, for the lasting voltage and current which are also important parameters of electricity grade, the voltage of PCF‐based LTED achieves a 42.3 times improvement, and the current improves as high as 47.5 times from 970 microamperes to 46.1 milliamperes (Figure 6h). The grade of generated electricity was remarkably upgraded. More importantly, the output power density achieved a 2010 times improvement from 2.01 µW to 4.04 mW (Figure 6g). The grade of generated electricity has been greatly improved, which could be applied to the more challenging occasions.

The calculated average electrical power output simultaneously acquired a high level of enhancement as high as 11.3%, which has never been reported before. As Equation (4) shows:

(4) $$Im_{p}=\frac{\Delta P}{P}\;=\frac{t\Delta P}{tP}\;=\frac{\Delta W}{W}\;=Im_{\mathrm{efficiency}}\;$$

The improvement of the total light‐thermal‐electric conversion efficiency (*Im*_efficiency_) increases the same value as the average electrical power output improves (*Im*_p_). Therefore, the total light‐thermal‐electric conversion efficiency based on PCFs‐LTED increases as high as 11.3% (Figure 6h), a significant value to promote the application of traditional thermoelectric devices.

The improvement could be extended to all thermoelectric devices. The device efficiency (*η*) could be calculated approximately by Equation (5):

(5) $$\eta \;=\frac{T_{h}-T_{c}}{T_{h}}\;\left[\frac{\sqrt{1+ZT}-1}{\sqrt{1+ZT}+\frac{T_{c}}{T_{h}}}\right]$$

where *T*_h_/*T*_c_ is the temperature of the hot/cold junction and *ZT* is the average figure of merit for the device over the temperature range *T*_c_ to *T*_h_.^[^ ^35^ ^]^ As shown, the device efficiency (*η*) increases with the *T*_h_, and decreases with *T*_c_. Therefore, when the heat releases from phase change materials, the temperature decrease is decayed and the temperature keeps at a relatively high level, the voltage, current and even power outputs could be notably upgraded, leading to an improved total light‐thermal‐electric conversion efficiency. Given that the energy comes from other sources such as waste heat rather than the sun or light, the phase‐change‐feedback heat could also improve the total energy conversion efficiency as the same. The phase‐change‐feedback method could be a remarkable solution to improve light‐thermal‐electric and even thermoelectric conversion efficiency.

In the last 10 years, the thermoelectric community has completed plenty of achievements to increase the thermoelectric conversion efficiency, whose value has reached a high level of about 15% (Figure 6i, Table S3, Supporting Information). Further improvement could be increasingly difficult. Herein, the phase‐change‐feedback strategy to improve energy conversion efficiency based on traditional thermoelectric devices could be vital. With a value of 11.3%, the thermoelectric conversion efficiency improvement could be more than 1.6%, which is fully competitive than the pure engineering on thermoelectric materials (Figure 6j). The phase‐change‐feedback strategy could be a promising route to aid the development of advanced thermoelectric devices.

## 3 Discussion and Conclusion

The main goal of this work is to fabricate a series of flexible multilayered PCFs by a simple origami process, and then verify its application in EMI shielding and light‐thermal‐electric conversion. Despite such a simple strategy, the origami strategy was demonstrated to be efficient to fabricate multifunctional nanocomposites. With just two or three simple folding processes, the EMI shielding efficiency could be tuned from 39.2 to 71.9 dB, which is capable of varying applications. The strategy is proved to be a general approach to developing novel multifunctional nanocomposites, as the light harvest performances of PCFs were simultaneously significantly enhanced by the origami process. The strategy could give some inspiration to the design and fabrication of other kinds of nanocomposites.

In addition, for the first time, the phase change process was reported able to improve the light‐thermal‐electric conversion efficiency based on traditional thermoelectric devices, with a total thermoelectric efficiency improvement as high as 11.3%. And the phase change process could significantly upgrade the quality of generated electricity. Both the average output voltage and current were improved remarkably with values of 27.3% and 23.8%, respectively. And the lasting voltage and current increase by 42.3 and 47.5 times, respectively. Such upgraded electricity could meet the requirements of more occasions. As the total thermoelectric conversion efficiency is difficult to improve, the design to introduce phase change materials into could be a facile and universal method to improve the thermoelectric conversion efficiency.

**Figure 7:**
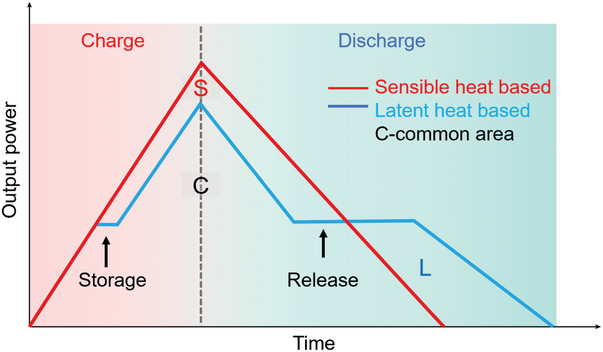
Power output of thermoelectric devices based on sensible heat and phase change nanocomposites.

Besides, there could be some improvement based on our work to further improve the total thermoelectric efficiency. Simulating the power output of thermoelectric devices based on sensible heat and phase change nanocomposites (**Figure** **7**), the calculated efficiency improvement could be written as Equation (6):

(6) $$I_{m}=\frac{\Delta \left(Latent\;heat-Sensible\;heat\right)}{Total\;sensible\;heat}\;=\frac{L\;-\;S}{S\;+\;C}\;$$

The S, L and C represent the corresponding area of sensible heat, latent heat and common area, respectively. When the latent heat exceeds the sensible heat, there will be efficiency improvements. On the contrary, the conversion efficiency is decreased. Therefore, the general methods to improve the total conversion efficiency are below: 1) Accelerate the charging process. In this regard, the curves of these two kinds are more closing, and the heat stored as the latent heat could be increased; 2) Extend the discharge time and more latent heat could be released (the power output is still larger than the control sample as shown); 3) Change the phase change materials into high‐temperature type, therefore the stored sensible heat will be reduced and latent heat increases. Moreover, with a high‐temperature phase change process, the quality of generated electricity could be further upgraded.

Overall, the work not only provides a general, simple strategy to fabricate multifunctional nanocomposites, but also comes up with a universal method to upgrade the generated electricity, the total light‐thermal‐electric and even thermoelectric conversion efficiency based on traditional thermoelectric devices. Both strategies show broad application prospects in electromagnetic shielding, energy storage and conversion.

## 4 Experimental Section

### Materials

Paraffin wax (PW, melting area: 41–44 °C) was purchased from Hangzhou Ruhr Tech Co. Ltd., China. Lithium chloride (LiCl), lithium fluoride (LiF), Dicumyl peroxide (DCP), 3‐hydroxytyramine dopamine (DA), and tris(hydroxymethyl) aminomethane (Tris) were purchased from Aladdin Chemicals Co., Ltd. (China). EVA was purchased from SK Global Chemical. Co., Ltd. Guangzhou Chemical Reagent Factory (China) provided hydrochloric acid (HCl, 37 wt%). MAX phase (Ti_3_AlC_2_, 99.7%) powder was supplied by Nanjing Mission New Materials Co., Ltd. (China). Fe_3_O_4_ nanoparticles solution (10–30 nm) was purchased from Macklin Industrial Co., Ltd.

### Characterization of Materials

The morphologies and microstructures of PCFs were observed with a field emission scanning electron microscope (SEM, Sirion 2000, FEI). The elemental composition was characterized using an energy‐dispersive X‐ray spectrometer (Xplore, Oxford Instrument Technology Co., Ltd., UK) coupled with SEM. The electrical conductivity was measured by a four‐point probe instrument (RTS‐8, Guangzhou Four‐Point Probe Technology Co. Ltd., China). A Fox 226s IR camera (fox Inc.) was employed to record the temperature distribution of PCFs in time. Phase change behavior of PW and PCFs were measured via a differential scanning calorimeter (DSC, Waters, America) between −20 to 80 °C at a scanning rate of 10.0 °C min^−1^ under a 40.0 mL min^−1^ nitrogen flow. The crystalline properties were examined by X‐ray diffraction (XRD, Smart Lab), with a scanning speed of 10° min^−1^ within 2–65°. The Fourier transform infrared spectroscopy (FT‐IR, Nicolet iS5, USA) was used to analyze the chemical structures. XPS was conducted on a spectrometer (VG ESCALAB MK II) with Al K*α* excitation radiation. The contact angle (CA) of the sample surface was characterized using automatic CA testing apparatus (JC2000, Shanghai Zhongchen Co., Ltd., China). The absorption spectra were monitored using a UV–vis‐NIR spectrophotometer (Lambda 750 S, PerkinElmer, USA). The voltages were supplied by a UTP 1306S (UNI‐T) DC power supply. An Agilent Technologies N5244A network analyzer was used to obtain electromagnetic parameters of PCFs in the frequency ranges of 8.2−12.4 GHz (X‐band) at room temperature. Before testing, the specimens were cut into 22.58 × 10.14 × 4 mm^3^ rectangular sheets.

### Fabrication of EVA/PW Composite

The PW/EVA composite was obtained by melt blending in a self‐developed biaxial eccentric rotor extruder (BERE) based on elongational rheology as reported in the previous work.^[^ ^36^ ^]^ And the ratio of PW, EVA, and DCP was 70:29:1. After the blends were obtained, the crosslinked PW/EVA films were produced followed by a hot‐pressing approach at 170 °C and 10 MPa for 5 min.

### Fabrication of PCFs composite

The EVA/PW film was coated by polydopamine in a tris buffer (PH = 8.5), in which the dopamine (concentration of 4 mg mL^−1^) polymerizes for 24 h with continuous stirring. The homogeneous MXene dispersion with a concentration of 12.0 mg mL^−1^ was synthesized according to the previous work^[^ ^37^ ^]^ (Figure S8, Supporting Information). Then, the EVA/PW film was coated by spray coating by two sequential layers of MXene and Fe_3_O_4_ (The images and characterization of Fe_3_O_4_ are present in Figure S9, Supporting Information), respectively. In all samples, the MXene content was 4.0 wt% of that of P film. Single‐layer F films with 1.0, 2.0, and 4.0 wt% Fe_3_O_4_ were labeled as F1, F2, F4, respectively. The multilayered PCFs were produced by the origami process. The F4 films were folded by 1/2/3 times to construct 2*F4, 4*F4, and 8*F4 PCFs, respectively. After the folding, the PCFs were put on a hot pan at 60 °C to link the adjacent layers. A control sample 4*[Fe_3_O_4_/MXene/polydopamine‐coated EVA film] (4*E4) was fabricated the same as 4*F4, the only difference is the basic film of 4*E4 is EVA film and that of 4*F4 is EVA/PW with the same thickness.

### Light‐Thermal‐Electric Conversion

The light‐thermal‐electric conversion was conducted under a simulated solar light generated by CEL‐S500L (AULTT, China). A commercial thermoelectric device (60 × 60 × 4 mm^3^, TEC) was put between the 4*F PCF (or 4*E4 control sample) and a cooling surface. In the experiment, the simulated solar light intensity was calibrated by an optical power meter (300–1100 nm, CEL‐FZ‐A Ceaulight, China). The temperature of the cooling surface was 10 ± 2 °C controlled by a low constant temperature bath. And the value of voltage and current were recorded by the data acquisition unit (Keysight DAQ6510).

## Conflict of Interest

The authors declare no conflict of interest.

## Supporting information

Supporting Information

Supplemental Video 1

Supplemental Video 2

Supplemental Video 3

## Data Availability

The data that support the findings of this study are available from the corresponding author upon reasonable request.
