# Supplementary material for: Upgrading Electricity Generation and Electromagnetic Interference Shielding Efficiency via Phase‐Change Feedback and Simple Origami Strategy
Source: Adv Sci (Weinh). 2023 Mar 22;10(14):2206835. doi: 10.1002/advs.202206835 (PMC10190587; doi:10.1002/advs.202206835)
Supplement: Supplementary file 1 — Supporting Information [file ADVS-10-2206835-s001.pdf]

ADVANCED SCIENCE
Open Access

Supporting Information

for *Adv. Sci.*, DOI 10.1002/advs.202206835

Upgrading Electricity Generation and Electromagnetic Interference Shielding Efficiency via Phase-Change Feedback and Simple Origami Strategy

Xinpeng Hu, Bingqing Quan, Chuanbiao Zhu, Haoye Wen, Mengjie Sheng, Shuang Liu, Xiaolong Li, Hao Wu, Xiang Lu and Jinping Qu**

Supporting Information *for*

Upgrading electricity generation and electromagnetic interference shielding efficiency via phase-change feedback and simple origami strategy

Xinpeng Hu [a, b], Bingqing Quan [a, b], Chuanbiao Zhu [a, b], Haoye Wen [a, b], Mengjie Sheng [a, b], Shuang Liu [a, b], Xiaolong Li [a, b], Hao Wu [a, b], Xiang Lu [a, b, *], Jinping Qu [a, b, *]

[a] Key Laboratory of Material Chemistry for Energy Conversion and Storage, Huazhong University of Science & Technology, Ministry of Education, Wuhan 430074, PR China

[b] Hubei Engineering Research Center for Biomaterials and Medical Protective Materials, Huazhong University of Science & Technology, Wuhan 430074, PR China

Corresponding author: Xiang Lu (luxiang@hust.edu.cn & luxiang_1028@163.com), Jinping Qu (jpqu@hust.edu.cn)

Supplementary Figures and Tables

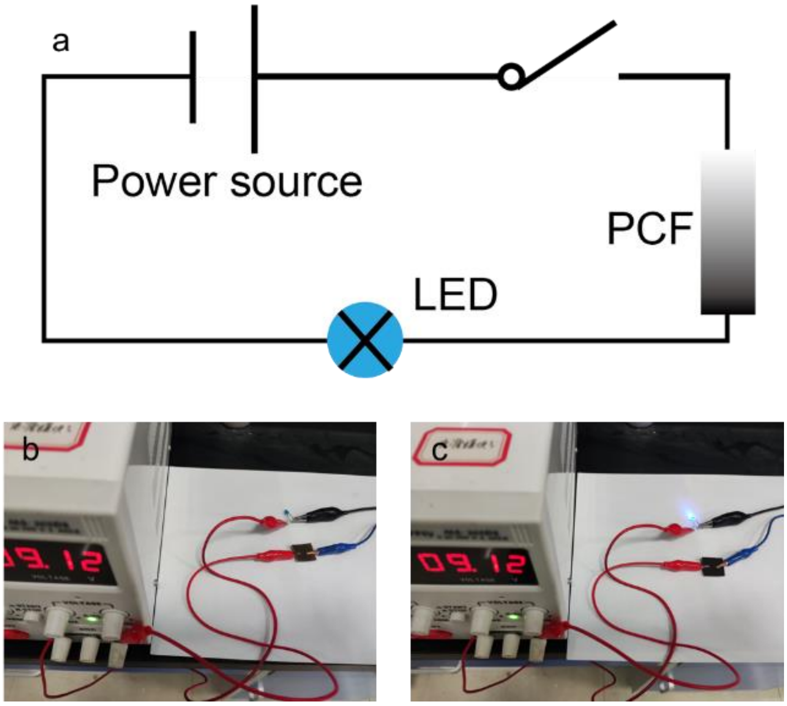

Figure S1. Conduction tests of PCFs. a) The circuit to drive the LED using PCFs as conductors. b) P film as conductor. c) F4 film as conductor.

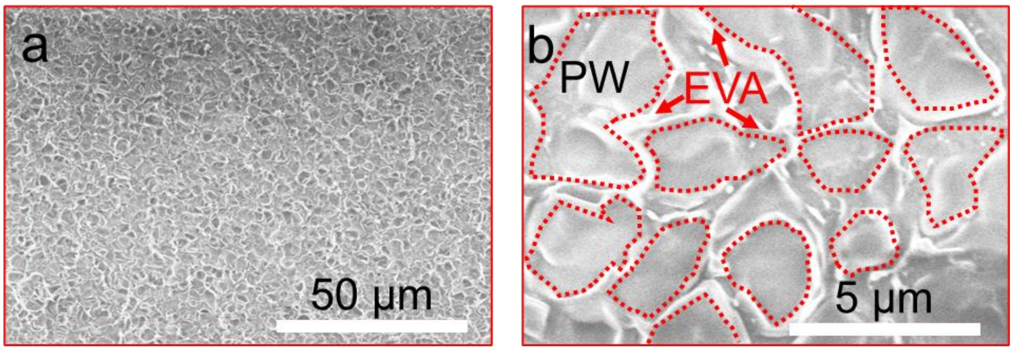

Figure S2. Fracture morphology of EVA/PW film.

The fracture surface of EVA/PW film shows a homogeneous morphology in which the PW is well dispersed into the EVA matrix (Figure S2a). In the morphology, the PW and EVA form a “sea-island” morphology where EVA is the sea and PW is the island, although the PW weight fraction is much higher than that of EVA. The PW are well constrained in the EVA matrix, because the EVA crosslinks into a continuous network. Besides, the interactions between EPDM and PW such as capillary force and Van der Waal force assist to keep the PW from leakage.

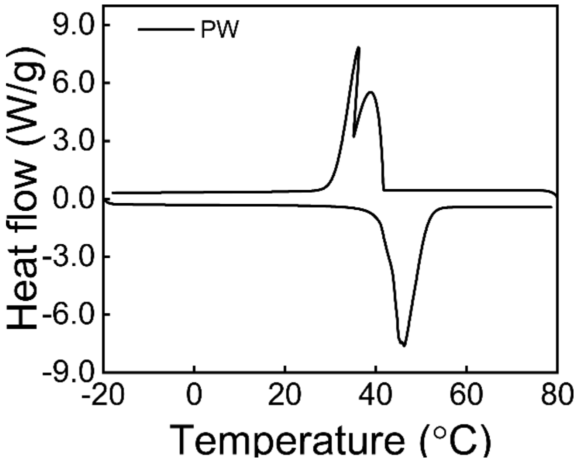

Figure S3. The differential scanning calorimetry curve of PW.

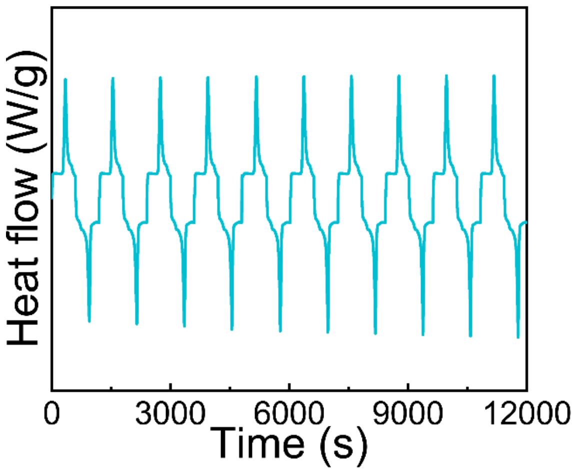

Figure S4. Differential scanning calorimetry curves of the F4 for 10 cycles.

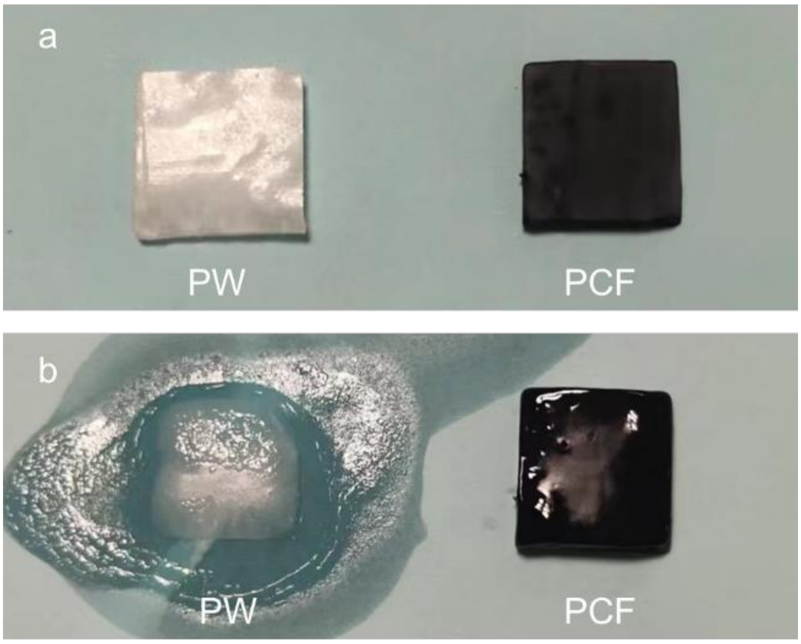

Figure S5. Shape stability of PCFs. a) The PW and 8* F4 PCF at room temperature. b) The PW and 8* F4 PCF beyond melting point.

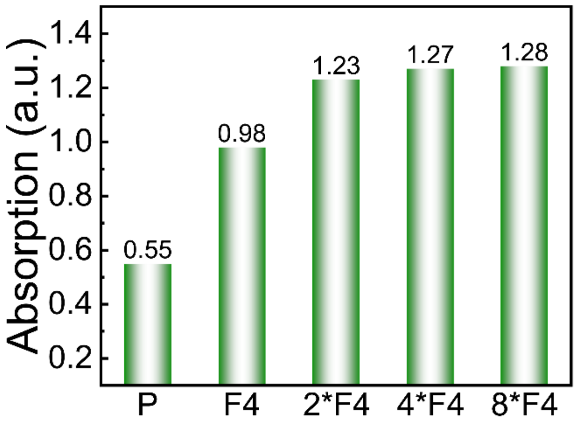

Figure S6. The average absorption rates of PCFs.

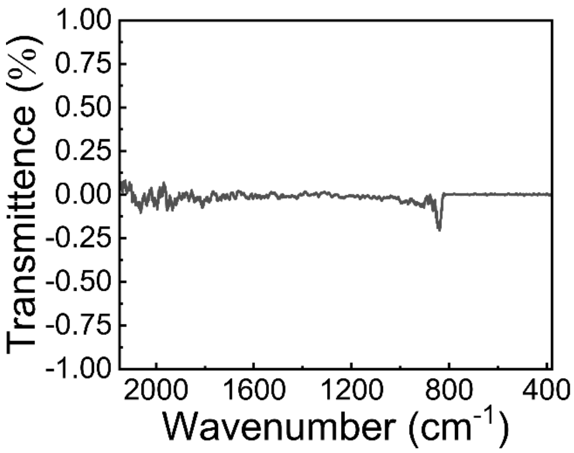

Figure S7. The transmittance fraction of light on 4*F4.

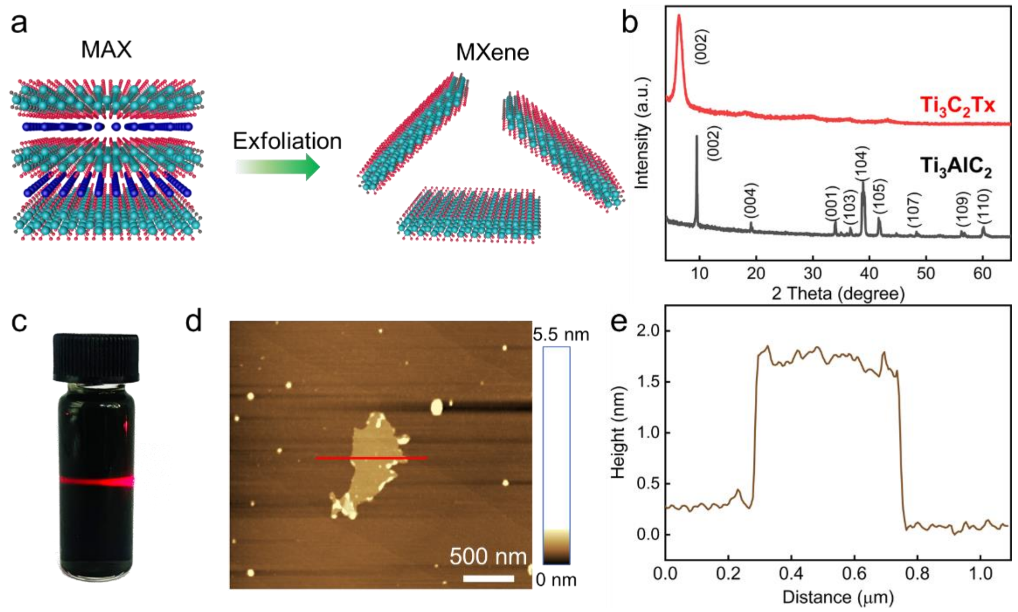

Figure S8. Sythesis of MXene and its characterizations. a) Illustration of MAX exfoliated into MXene. b) XRD of MAX and $Ti_3C_2T_X$ MXene. c) Tyndall effect of MXene dispersion. d) AFM of MXene nanosheet. e) corresponding thickness of MXene sheet.

Successful synthesis of high-quality MXene sheets is verified by X-ray diffraction (XRD), Atomic Force Microscopy (AFM), and Tyndall effect[1]. As reported, the selective removal of Al layers from Ti_3AlC_2 MAX phase with a LiF/HCl solution yields clay-like unexfoliated $Ti_3C_2T_X$ MXene (Figure S8a). This is proved by the leftward shifted (002) peak and the absent or weakened characteristic peaks of Ti_3AlC_2 (Figure S8b). The few-layer $Ti_3C_2T_X$ nanosheets was samultaneously proved by Tyndall effect and AFM cahracterization. As the laser light enters the MXene dispersion, there is a distinct beam in the dispersion (Figure S8c), revealing that the nanopartical size in the dispersion ranges from 1-100nm[2]. In addition, the AFM images reveals that the thickness of MXene nanosheets is below 5nm, the calculated average thickness is ~ 1.75nm (Figure S8d,e). All these results demonstrate that a high-quality MXene dispersion was synthesized.

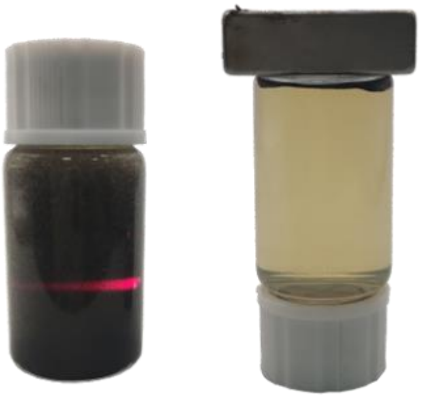

Figure S9. The characterizations of Fe_3O_4.

As shown by Figure S9, the size of Fe_3O_4 nanoparticles was demonstrated to be in the range of 1-100 nm, as there is obvious Tydall effect existing in the dispersion. Besides, after put a magnet under the dispersion and turn around, the Fe_3O_4 nanoparticles are adhesive to the surface closed to the magnet, revealing that the Fe_3O_4 nanoparticles still possess excellent magnetism.

Table S1. Comparison for EMI shielding performances of different MXene-based EMI shielding films at X-band.

Filler	Matrix	Content (wt %)	SE/dB	Ref
MXene	ANF	91	34.7	[3]
MXene	CNF	44	39.6	[4]
MXene	ANF	40	24.5	[5]
MXene	PEDOT:PSS	88	42.1	[6]
MXene/CF	Epoxy	25	14.06	[7]
MXene	chitosan	75	34.7	[8]
MXene	PI/ANF	35	48.9	[9]
MXene/CNT	CNF	80	23.4	[10]
MXene	TOCNF	50	39.6	[11]
MXene	r-CNFs	70	42.7	[12]
MXene	SA	90	43.9	[13]
MXene	CNF	80	25.8	[14]
MXene	PVA	40	40	[15]
MXene/Fe_3O_4/CNTs	-----	100	49	[16]
MXene/Fe_3O_4	EVA/PW	8	71.9	This work

Table S2. Comparison in terms of output power density for PCM-based LTEG reported in previous research.

PCM	Irradiation power (kW/m^2)	Output power density (W/m^2)	Ref
PEG	4	~6.3	[17]
	4	~7.5	[17]
	8	~15.8	[17]
	8	~22.5	[17]
PEG	3	~1.4	[18]
	4	~2.0	[18]
	8	~2.3	[18]
n-eicosane	0.5	0.03	[19]
PEG	8	~5.1	[20]
PEG	1	~0.24	[21]
PEG	8	~2.19	[22]
CA	0.69	32.8	[23]
PEG	0.78	40.3	[24]
PW	1	55.2	This work

Table S3. Summarization of thermoelectric conversion efficiency of typical thermoelectric materials from 2013 to 2022.

References	Year	Thermoelectric conversion efficiency (%)
[25]	2013	5.29
[26]	2013	12.5
[27]	2014	5.4
[28]	2014	10.9
[29]	2015	8.5
[30]	2015	14
[31]	2016	6
[32]	2016	12
[33]	2017	4.5
[34]	2017	8.5
[35]	2017	9.3
[36]	2017	12
[37]	2018	3.3
[38]	2018	5
[39]	2018	12
[40]	2019	2.83
[41]	2019	3
[42]	2019	7.8
[43]	2019	9.1
[44]	2019	9.15
[45]	2019	12.3
[46]	2020	6.6
[47]	2020	8.3
[48]	2020	9.12
[49]	2020	10.5

[50]	2020	11
[51]	2020	12
[52]	2020	12.4
[53]	2021	4.2
[54]	2021	7.3
[55]	2021	7.4
[56]	2021	7.8
[57]	2021	8.7
[58]	2021	12.3
[59]	2021	12.4
[60]	2021	13
[61]	2021	14
[62]	2022	5.37
[63]	2022	10
[64]	2022	12.2
[65]	2022	12.8
[66]	2022	13.6
[67]	2022	14.56

References

[1] S. Gong, X. Sheng, X. Li, M. Sheng, H. Wu, X. Lu, J. Qu, *Adv. Funct. Mater.* **2022**, *32*, 2200570, https://doi.org/10.1002/adfm.202200570.

[2] Z. Ma, S. Kang, J. Ma, L. Shao, Y. Zhang, C. Liu, A. Wei, X. Xiang, L. Wei, J. Gu, *ACS Nano* **2020**, *14* (7), 8368, https://doi.org/10.1021/acsnano.0c02401.

[3] H. W. Wei, M. Q. Wang, W. H. Zheng, Z. X. Jiang, Y. D. Huang, *Ceram. Int.* **2020**, *46* (5), 6199, https://doi.org/10.1016/j.ceramint.2019.11.087.

[4] B. Zhou, Z. Zhang, Y. Li, G. Han, Y. Feng, B. Wang, D. Zhang, J. Ma, C. Liu, *ACS Appl. Mater. Interfaces* **2020**, *12* (4), 4895, https://doi.org/10.1021/acsami.9b19768.

[5] C. X. Lei, Y. Z. Zhang, D. Y. Liu, K. Wu, Q. Fu, *ACS Appl. Mater. Interfaces* **2020**, *12* (23), 26485, https://doi.org/10.1021/acsami.0c07387.

[6] R. Liu, M. Miao, Y. Li, J. Zhang, S. Cao, X. Feng, *ACS Appl. Mater. Interfaces* **2018**, *10* (51), 44787, https://doi.org/10.1021/acsami.8b18347.

[7] S. Lee, D. Park, Y. Cho, J. Lee, J. Kim, *Synth. Met.* **2022**, *291*, 117183, https://doi.org/10.1016/j.synthmet.2022.117183.

[8] F. Liu, Y. Li, S. Hao, Y. Cheng, Y. Zhan, C. Zhang, Y. Meng, Q. Xie, H. Xia, *Carbohydr Polym* **2020**, *243*, 116467, https://doi.org/10.1016/j.carbpol.2020.116467.

[9] L. Zhuo, Y. Cai, D. Shen, P. Gou, M. Wang, G. Hu, F. Xie, *Chem. Eng. J.* **2023**, *451*, 138808, https://doi.org/10.1016/j.cej.2022.138808.

[10] W. Cao, C. Ma, S. Tan, M. Ma, P. Wan, F. Chen, *Nano-Micro Lett.* **2019**, *11* (1), https://doi.org/10.1007/s40820-019-0304-y.

[11] Z. Zhan, Q. Song, Z. Zhou, C. Lu, *J. Mater. Chem. C* **2019**, *7* (32), 9820, https://doi.org/10.1039/c9tc03309b.

[12] C. Cui, C. Xiang, L. Geng, X. X. Lai, R. H. Guo, Y. Zhang, H. Y. Xiao, J. W. Lan, S. J. Lin, S. X. Jiang, *J. Alloys Compd.* **2019**, *788*, 1246, https://doi.org/10.1016/j.jallcom.2019.02.294.

[13] Z. H. Zhou, J. Z. Liu, X. X. Zhang, D. Tian, Z. Y. Zhan, C. H. Lu, *Adv. Mater. Interfaces* **2019**, *6* (6), 1802040, https://doi.org/10.1002/admi.201802040.

[14] W. Cao, F. Chen, Y. Zhu, Y. Zhang, Y. Jiang, M. Ma, F. Chen, *ACS Nano* **2018**, *12* (5), 4583, https://doi.org/10.1021/acsnano.8b00997.

[15] Y. Zhang, K. Ruan, J. Gu, *Small* **2021**, *17* (42), e2101951, https://doi.org/10.1002/smll.202101951.

[16] H. Liu, Z. Wang, Y. Yang, S. Wu, C. Wang, C. You, N. Tian, *J. Mater. Sci. Technol.* **2022**, *130*, 75, https://doi.org/10.1016/j.jmst.2022.05.009.

[17] J. Yang, L.-S. Tang, R.-Y. Bao, L. Bai, Z.-Y. Liu, W. Yang, B.-H. Xie, M.-B. Yang, *J. Mater. Chem. A* **2016**, *4* (48), 18841, https://doi.org/10.1039/c6ta08454k.

[18] *Chem. Eng. J.* **2017**, *315*, 481, https://doi.org/10.1016/j.cej.2017.01.045.

[19] M. S. Kim, M. K. Kim, H. R. Ahn, Y. J. Kim, Ieee, in *28th IEEE International Conference on Micro Electro Mechanical Systems (MEMS)* **2015**, 1114-1117.

[20] J. Yang, L.-S. Tang, L. Bai, R.-Y. Bao, Z. Liu, B.-H. Xie, M.-B. Yang, W. Yang, *ACS Sustainable Chem. Eng.* **2018**, *6* (5), 6761, https://doi.org/10.1021/acssuschemeng.8b00565.

[21] F. Xue, X. Z. Jin, X. Xie, X. D. Qi, J. H. Yang, Y. Wang, *Nanoscale* **2019**, *11* (40), 18691, https://doi.org/10.1039/c9nr07273j.

[22] J. Yang, P. Yu, L. S. Tang, R. Y. Bao, Z. Y. Liu, M. B. Yang, W. Yang, *Nanoscale* **2017**, *9* (45), 17704, https://doi.org/10.1039/c7nr05449a.

[23] Y. Jeyashree, Y. Sukhi, A. Vimala Juliet, S. Lourdu Jame, S. Indirani, *Mater. Sci. Semicond. Process.* **2020**, *107*, 104782, https://doi.org/10.1016/j.mssp.2019.104782.

[24] D. Liu, C. Lei, K. Wu, Q. Fu, *ACS Nano* **2020**, *14* (11), 15738, https://doi.org/10.1021/acsnano.0c06680.

[25] C. Hadjistassou, E. Kyriakides, J. Georgiou, *Energy Convers. Manage.* **2013**, *66*, 165, https://doi.org/10.1016/j.enconman.2012.07.030.

[26] W. Liu, K. C. Lukas, K. McEnaney, S. Lee, Q. Zhang, C. P. Opeil, G. Chen, Z. Ren, *Energy Environ. Sci.* **2013**, *6* (2), 552, https://doi.org/10.1039/c2ee23549h.

[27] D. Yoo, J. Kim, J. H. Kim, *Nano Res.* **2014**, *7* (5), 717, https://doi.org/10.1007/s12274-014-0433-z.

[28] X. Sun, X. Liang, G. Shu, H. Tian, H. Wei, X. Wang, *Energy* **2014**, *77*, 489, https://doi.org/10.1016/j.energy.2014.09.032.

[29] D. Kraemer, J. Sui, K. McEnaney, H. Zhao, Q. Jie, Z. F. Ren, G. Chen, *Energy Environ. Sci.* **2015**, *8* (4), 1299, https://doi.org/10.1039/c4ee02813a.

[30] E. Hazan, O. Ben-Yehuda, N. Madar, Y. Gelbstein, *Adv. Energy Mater.* **2015**, *5* (11), 201500272, https://doi.org/10.1002/aenm.201500272.

[31] F. Hao, P. Qiu, Y. Tang, S. Bai, T. Xing, H.-S. Chu, Q. Zhang, P. Lu, T. Zhang, D. Ren, J. Chen, X. Shi, L. Chen, *Energy Environ. Sci.* **2016**, *9* (10), 3120, https://doi.org/10.1039/c6ee02017h.

[32] Q. Zhang, E. K. Chere, Y. Wang, H. S. Kim, R. He, F. Cao, K. Dahal, D. Broido, G. Chen, Z. Ren, *Nano Energy* **2016**, *22*, 572, https://doi.org/10.1016/j.nanoen.2016.02.040.

[33] F. Meng, L. Chen, Y. Feng, B. Xiong, *Energy* **2017**, *135*, 83, https://doi.org/10.1016/j.energy.2017.06.086.

[34] X. Tan, L. Wang, H. Shao, S. Yue, J. Xu, G. Liu, H. Jiang, J. Jiang, *Adv. Energy Mater.* **2017**, *7* (18), 201700076, https://doi.org/10.1002/aenm.201700076.

[35] Q. Zhang, Z. Zhou, M. Dylla, M. T. Agne, Y. Pei, L. Wang, Y. Tang, J. Liao, J. Li, S. Bai, W. Jiang, L. Chen, G. J. Snyder, *Nano Energy* **2017**, *41*, 501, https://doi.org/10.1016/j.nanoen.2017.10.003.

[36] Q. Zhang, J. Liao, Y. Tang, M. Gu, C. Ming, P. Qiu, S. Bai, X. Shi, C. Uher, L. Chen, *Energy Environ. Sci.* **2017**, *10* (4), 956, https://doi.org/10.1039/c7ee00447h.

[37] B. Aravind, G. K. S. Raghuram, V. R. Kishore, S. Kumar, *Energy Convers. Manage.* **2018**, *156*, 224, https://doi.org/10.1016/j.enconman.2017.11.021.

[38] R. Deng, X. Su, S. Hao, Z. Zheng, M. Zhang, H. Xie, W. Liu, Y. Yan, C. Wolverton, C. Uher, M. G. Kanatzidis, X. Tang, *Energy Environ. Sci.* **2018**, *11* (6), 1520, https://doi.org/10.1039/c8ee00290h.

[39] P. Jood, M. Ohta, A. Yamamoto, M. G. Kanatzidis, *Joule* **2018**, *2* (7), 1339, https://doi.org/10.1016/j.joule.2018.04.025.

[40] Y. Choi, A. Negash, T. Y. Kim, *Energy Convers. Manage.* **2019**, *197*, 111902, https://doi.org/10.1016/j.enconman.2019.111902.

[41] X. Wang, H. Wang, W. Su, J. Zhai, T. Wang, T. Chen, F. Mehmood, C. Wang, *Renewable Energy* **2019**, *131*, 606, https://doi.org/10.1016/j.renene.2018.07.067.

[42] X. Lu, Q. Zhang, J. Liao, H. Chen, Y. Fan, J. Xing, S. Gu, J. Huang, J. Ma, J. Wang, L. Wang, W. Jiang, *Adv. Energy Mater.* **2020**, *10* (2), 201902986, https://doi.org/10.1002/aenm.201902986.

[43] P. Qiu, T. Mao, Z. Huang, X. Xia, J. Liao, M. T. Agne, M. Gu, Q. Zhang, D. Ren, S. Bai, X. Shi, G. J. Snyder, L. Chen, *Joule* **2019**, *3* (6), 1538, https://doi.org/10.1016/j.joule.2019.04.010.

[44] G. Nie, W. Li, J. Guo, A. Yamamoto, K. Kimura, X. Zhang, E. B. Isaacs, V. Dravid, C. Wolverton, M. G. Kanatzidis, S. Priya, *Nano Energy* **2019**, *66*, https://doi.org/10.1016/j.nanoen.2019.104193.

[45] S. Perumal, M. Samanta, T. Ghosh, U. S. Shenoy, A. K. Bohra, S. Bhattacharya, A. Singh, U. V.

Waghmare, K. Biswas, *Joule* **2019**, *3* (10), 2565, https://doi.org/10.1016/j.joule.2019.08.017.
[46] B. Zhu, X. X. Liu, Q. Wang, Y. Qiu, Z. Shu, Z. T. Guo, Y. Tong, J. Cui, M. Gu, J. Q. He, *Energy Environ. Sci.* **2020**, *13* (7), 2106, https://doi.org/10.1039/d0ee01349h.
[47] J. Yu, Y. Xing, C. Hu, Z. Huang, Q. Qiu, C. Wang, K. Xia, Z. Wang, S. Bai, X. Zhao, L. Chen, T. Zhu, *Adv. Energy Mater.* **2020**, *10* (25), https://doi.org/10.1002/aenm.202000888.
[48] S. Lv, M. Liu, W. He, X. Li, W. Gong, S. Shen, *Energy Convers. Manage.* **2020**, *207*, 112516, https://doi.org/10.1016/j.enconman.2020.112516.
[49] Y. Xing, R. Liu, J. Liao, C. Wang, Q. Zhang, Q. Song, X. Xia, T. Zhu, S. Bai, L. Chen, *Joule* **2020**, *4* (11), 2475, https://doi.org/10.1016/j.joule.2020.08.009.
[50] R. A. Kishore, A. Nozariasbmarz, B. Poudel, S. Priya, *ACS Appl. Mater. Interfaces* **2020**, *12* (9), 10389, https://doi.org/10.1021/acsami.9b21299.
[51] W. Li, B. Poudel, A. Nozariasbmarz, R. Sriramdas, H. Zhu, H. B. Kang, S. Priya, *Adv. Energy Mater.* **2020**, *10* (38), 202001924, https://doi.org/10.1002/aenm.202001924.
[52] Q. Zhu, Z. Ren, *Energy* **2020**, *191*, https://doi.org/10.1016/j.energy.2019.116599.
[53] B. Cai, H.-L. Zhuang, J. Pei, B. Su, J.-W. Li, H. Hu, Y. Jiang, J.-F. Li, *Nano Energy* **2021**, *85*, 106040, https://doi.org/10.1016/j.nanoen.2021.106040.
[54] Z. Liu, N. Sato, W. Gao, K. Yubuta, N. Kawamoto, M. Mitome, K. Kurashima, Y. Owada, K. Nagase, C.-H. Lee, J. Yi, K. Tsuchiya, T. Mori, *Joule* **2021**, *5* (5), 1196, https://doi.org/10.1016/j.joule.2021.03.017.
[55] G. Bai, Y. Yu, X. Wu, J. Li, Y. Xie, L. Hu, F. Liu, M. Wuttig, O. Cojocaru-Miredin, C. Zhang, *Adv. Energy Mater.* **2021**, *11* (37), 202102012, https://doi.org/10.1002/aenm.202102012.
[56] T. Xing, Q. Song, P. Qiu, Q. Zhang, M. Gu, X. Xia, J. Liao, X. Shi, L. Chen, *Energy Environ. Sci.* **2021**, *14* (2), 995, https://doi.org/10.1039/d0ee02791j.
[57] S. E. Yang, F. Kim, F. Ejaz, G. S. Lee, H. Ju, S. Choo, J. Lee, G. Kim, S.-h. Jung, S. Ahn, H. G. Chae, K. T. Kim, B. Kwon, J. S. Son, *Nano Energy* **2021**, *81*, 105638, https://doi.org/10.1016/j.nanoen.2020.105638.
[58] B. Jiang, Y. Yu, J. Cui, X. Liu, L. Xie, J. Liao, Q. Zhang, Y. Huang, S. Ning, B. Jia, B. Zhu, S. Bai, L. Chen, S. J. Pennycook, J. He, *Science* **2021**, *371* (6531), 830, https://doi.org/10.1126/science.abe1292.
[59] X. Lou, S. Li, X. Chen, Q. Zhang, H. Deng, J. Zhang, D. Li, X. Zhang, Y. Zhang, H. Zeng, G. Tang, *ACS Nano* **2021**, *15* (5), 8204, https://doi.org/10.1021/acsnano.1c01469.
[60] K. Song, D. Yin, H. Song, P. Schiavone, X. Wu, L. Yuan, *Energy* **2022**, *239*, 122440, https://doi.org/10.1016/j.energy.2021.122440.
[61] Z. Bu, X. Zhang, B. Shan, J. Tang, H. Liu, Z. Chen, S. Lin, W. Li, Y. Pei, *Sci. Adv.* **2021**, *7* (19), https://doi.org/10.1126/sciadv.abf2738.
[62] Y.-K. Zhu, Y. Sun, J. Zhu, K. Song, Z. Liu, M. Liu, M. Guo, X. Dong, F. Guo, X. Tan, B. Yu, W. Cai, J. Jiang, J. Sui, *Small* **2022**, *18* (23), 202201352, https://doi.org/10.1002/smll.202201352.
[63] X. L. Shi, W. D. Liu, M. Li, Q. Sun, S. D. Xu, D. Du, J. Zou, Z. G. Chen, *Adv. Energy Mater.* **2022**, *12* (20), 202200670, https://doi.org/10.1002/aenm.202200670.
[64] B. Jia, Y. Huang, Y. Wang, Y. Zhou, X. Zhao, S. Ning, X. Xu, P. Lin, Z. Chen, B. Jiang, J. He, *Energy Environ. Sci.* **2022**, *15* (5), 1920, https://doi.org/10.1039/d1ee03883d.
[65] C. Xu, Z. Liang, W. Ren, S. Song, F. Zhang, Z. Ren, *Adv. Energy Mater.* **2022**, 202202392, https://doi.org/10.1002/aenm.202202392.
[66] J. Cao, X. Y. Tan, N. Jia, J. Zheng, S. W. Chien, H. K. Ng, C. K. I. Tan, H. Liu, Q. Zhu, S. Wang, G. Zhang, K. Chen, Z. Li, L. Zhang, J. Xu, L. Hu, Q. Yan, J. Wu, A. Suwardi, *Nano Energy* **2022**, *96*,

107147, https://doi.org/10.1016/j.nanoen.2022.107147.
[67] B. Nan, G. Xu, W.-M. Liu, Q. Yang, B. Zhang, Y. Dong, J. Tie, T. Guo, X. Zhou, *Mater. Today Commun.* **2022**, *31*, 103343, https://doi.org/10.1016/j.mtcomm.2022.103343.
